# Microstructure Evolution and Mechanical Properties of Fe-25Ni-15Cr Alloy During Cumulative Cold-Drawing Deformation Process

**DOI:** 10.3390/nano15221717

**Published:** 2025-11-13

**Authors:** Yunfei Zhang, Zhen Zhang, Wei Chen, Zhongjie Tian, Xueliang An, Yang Zhang, Zhongwu Zhang

**Affiliations:** 1 College of Materials Science and Chemical Engineering, Harbin Engineering University, Harbin 150001, China; zhangyunfei@hbisco.com; 2 HBIS Group, HBIS Materials Technology Research Institute, Shijiazhuang 050023, China; zhangzhen@hbisco.com (Z.Z.); chenwei4400@hbisco.com (W.C.); tianzhongjie@hbisco.com (Z.T.); anxueliang@hbisco.com (X.A.)

**Keywords:** Fe-25Ni-15Cr alloy, cold drawing, deformation amount, mechanical properties

## Abstract

In this study, we fabricated Fe-25Ni-15Cr alloy rods via vacuum induction melting, electroslag remelting, forging, hot rolling, and annealing. We systemically investigated the influence of varying cold-drawing deformation levels (10–60%) on microstructure evolution and mechanical properties, which were characterized by a variety of multi-scale characterization techniques, including optical microscopy, scanning electron microscopy, X-ray diffraction, and transmission electron microscopy. The results show that when the cumulative deformation amount is less than 30%, the hardness, tensile strength, and yield strength increase significantly with the increase in deformation amount, while the elongation continues to decline; when the cumulative deformation amount exceeds 30%, the rates of increase in hardness and strength decrease significantly; and when the deformation amount increases to 50%, dislocation density accumulates preferentially at the grain boundaries and forms a cellular substructure, while the texture orientation gradually stabilizes from random distribution to the <111> direction. This alloy rod exhibits three strengthening mechanisms during cold drawing: grain refinement, second-phase precipitation, and work hardening. A predictive model for tensile strength is derived through theoretical calculations. This work has guiding significance for establishing a cold-drawing process window without intermediate annealing.

## 1. Introduction

The Fe-25Ni-15Cr alloy is a precipitation-hardening high-temperature alloy. Due to its excellent high-temperature strength, fatigue resistance, and corrosion resistance, it is widely used in key hot end components such as fasteners for aircraft engines and turbine blades for gas turbines [1,2,3,4,5,6,7,8]. With the continuous development of the aviation industry, aviation equipment is gradually moving towards higher thrust-to-weight ratios, higher temperature resistance, and longer service life, which impose higher requirements on the comprehensive performance of alloys [9,10,11,12,13,14,15].

In the process of manufacturing key components such as fasteners for aero engines, as the main processing method for small-sized bars, cold drawing can prevent surface oxidation and enhance mechanical properties. The Fe-25Ni-15Cr alloy bar needs to achieve the dual requirements of dimensional accuracy and mechanical properties through multiple cold-drawing processes. As a core process parameter, cumulative deformation directly affects the dislocation density, grain orientation, and second-phase distribution within the material and thus determines the strength, plasticity, and fatigue performance of the finished product.

Traditional single-pass deformation studies struggle to reveal the evolution laws of complex mechanisms such as microstructural evolution and dislocation proliferation in multi-pass deformation. In actual production, blindly increasing the single-pass deformation amount makes the material prone to cracking risk [16]. Therefore, a systematic study of the cumulative deformation regulation law on microstructures and properties not only helps to break through the strength bottleneck of the existing cold-drawing process but also provides theoretical support for formulating the energy-saving process of “high cumulative deformation–no annealing times”, which has significant engineering value for reducing aviation component manufacturing costs and improving the material utilization rate.

However, current studies on the production process of Fe-25Ni-15Cr alloy mainly focus on the influence of smelting, hot working (such as forging and rolling), and solution aging heat treatment on the mechanical properties [3,17,18,19,20,21,22], while there is relatively little research on the cold-drawing process. Qin et al. [23] investigated the effects of different single-pass deformation amounts on the microstructure and properties of Fe-25Ni-15Cr alloy. By combining this with a reasonable heat treatment system, they ultimately obtained a cold-drawn Fe-25Ni-15Cr bar with a strength of 1100 MPa. Liu et al. [24] found that ultimate tensile strength could be enhanced by increasing the drawing deformation amount. The cold plastic deformation mechanism of alloys is jointly regulated by dislocation slip and twinning behavior [25,26,27]. Huang et al. [28] explored the influence mechanism of twinning on the strength–plasticity relationship of Fe-25Ni-15Cr alloy under different pre-strain conditions, revealing a unique twinning–detwinning transformation process under high pre-strain conditions. Some other studies have found that introducing deformed nanotwins into metallic materials can significantly enhance their strength while maintaining good plasticity [29,30,31,32,33]. Although previous studies have conducted preliminary explorations on the cold deformation behavior of Fe-25Ni-15Cr alloy, research on the cumulative cold-drawing deformation behavior has not yet been reported; therefore, in this paper, we conduct a systematic study on the continuous cold drawing of Fe-25Ni-15Cr alloy without annealing, exploring the influence of different cumulative cold-drawing deformation amounts on the microstructure and room-temperature mechanical properties, and establishing a prediction model of the cold-drawing deformation amount and tensile strength. This provides a theoretical basis for reducing the production costs of Fe-25Ni-15Cr alloy cold-drawn bars and establishing a “high cumulative deformation amount–no annealing” cold-drawing process.

## 2. Materials and Methods

The Fe-25Ni-15Cr alloy used in this experiment was produced by vacuum induction melting and electroslag remelting. The electroslag ingot was billeted by a fast-forging machine and then hot rolled into a φ 16 mm bar. Subsequently, the hot-rolled bars were annealed at 850 °C for 0.5 h, followed by water cooling. Then, the annealed rod was subjected to peeling treatment, with the diameter of the rod peeled from φ 16 mm to φ 15 mm to obtain the billet for the cold-drawing experiments. The microstructure was observed using a Zeiss Olympus DSX 1000 optical microscope produced in Jena, Germany.

Subsequently, a tensile test was conducted on the cold-drawn billet. The Z600E-600N electronic tensile testing machine produced by Zwick in Ulm, Germany was used for testing, with a strain rate of 2.5 × 10^−3^ s^−1^. The tensile specimens were prepared through a CNC lathe in accordance with the GB/T 228.1-2010 standard [34]. The cumulative deformation amount and its corresponding specifications were 10% (φ 14.2 mm), 20% (φ 13.4 mm), 30% (φ 12.7 mm), 40% (φ 12 mm), 50% (φ 11.3 mm), and 60% (φ 10.7 mm), respectively.

**Figure 1:**
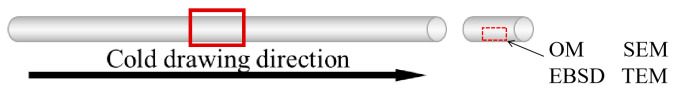
Schematic diagram of sampling location and microstructure detection location.

The evolution process of the microstructure and properties of the alloy with the change in the cumulative deformation amount was studied. A 20 mm long cylindrical section was cut from the cold-drawn billet and longitudinally split along its central axis into two halves. Please refer to Figure 1 for the specific sampling and testing locations. After mechanical grinding and polishing, the sample was etched in a solution of 5 g copper chloride (Made by Wujiang Shengfeng Chemical Co., Ltd. in Suzhou, China) + 100 mL hydrochloric acid (Made by Tianjin Kermel Chemical Reagent Co., Ltd. in Tianjin, China) + 100 mL anhydrous ethanol (Made by Beijing Chemical Works in Beijing, China) for 60 s. Samples for scanning electron microscopy (SEM) measurement were prepared by electropolishing and electrolysis, and the equipment used was the S-3400N scanning electron microscope produced by Hitachi, Tokyo, Japan. EDS energy spectrum analysis was utilized to analyze the composition of the precipitates. The acceleration voltage set to 20 KV and a secondary electron measurement mode, which was used to study the microstructure of the materials.

The texture and dislocation density of the material were studied using an oriented imaging microscope (EBSD) on a Hitachi S-3400N scanning electron microscope produced in Tokyo, Japan. EBSD samples were also prepared using electropolishing and electrolytic treatment. EBSD and SEM samples were subjected to electropolishing and electrolytic treatment after mechanical polishing; the former was carried out at a voltage of 25 V for 12 s, while the latter was conducted at 4.8 volts for 15 s. The microstructure and dislocation evolution process of the material were systematically studied using a Tecnai F30 transmission electron microscope produced by FEI Corporation in Hillsboro, OR, USA under an acceleration voltage of 200 kV. Specimens for TEM observation were first cut into thin slices approximately 300 μm thick, then mechanically ground to a thickness of 40–50 μm and punched into disks of Φ 3 mm. The disks were thinned using a double-jet electrolytic polisher. The electrolyte contained 10% perchloric acid (Made by Tianjin Xinyuan Chemical Co., Ltd. in Tianjin, China) and 90% ethanol (Made by Beijing Chemical Works in Beijing, China), maintained at −30 ± 1 °C, with a voltage of 25 V. Finally, an ion miller (Made by Futai Microscience Instrument Co., Ltd. in Shanghai, China) Gatan 695, operating at a voltage of 3.0 V and an incidence angle of ±3°) was used to expand the thinning area.

The Rockwell hardness (HRC) was measured using the Wilson R4/T Rockwell hardness tester produced by ITW Corporation in Glenview, IL, USA. At least 10 measurements were conducted at different points on each sample to obtain the average hardness value. X-ray fluorescence spectrometry analysis was conducted using the Dutch PANalytical AxiosmAX, Almelo, The Netherlands.

## 3. Results

### 3.1. Cold-Drawn Billet

**Figure 2:**
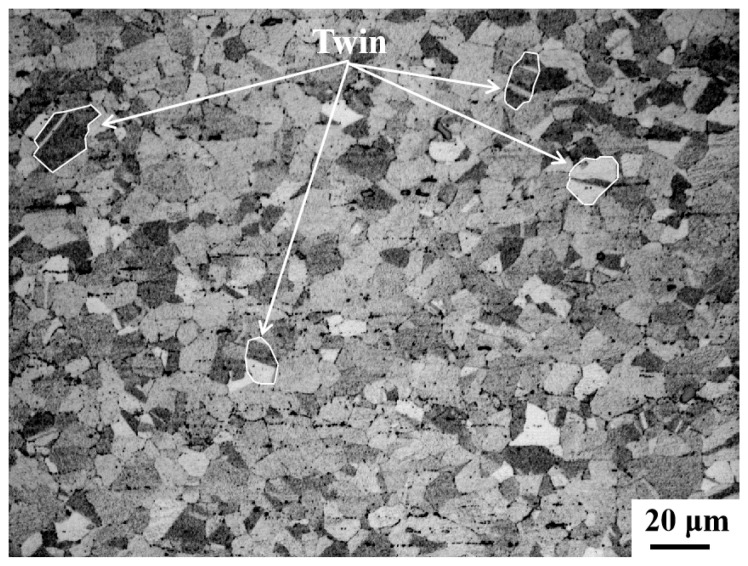
Metallographic microstructure of Fe-25Ni-15Cr alloy cold-drawn billet.

The chemical composition of the cold-drawn billet is shown in Table 1. As shown in Figure 2, the grains are equiaxed with a few twins present. The overall structure is uniform and fine, with an average grain size of 12.98 ± 1 μm. After testing, the tensile strength of the cold-drawn experimental billet is 673 MPa.

### 3.2. Room-Temperature Hardness and Tensile Change

**Figure 3:**
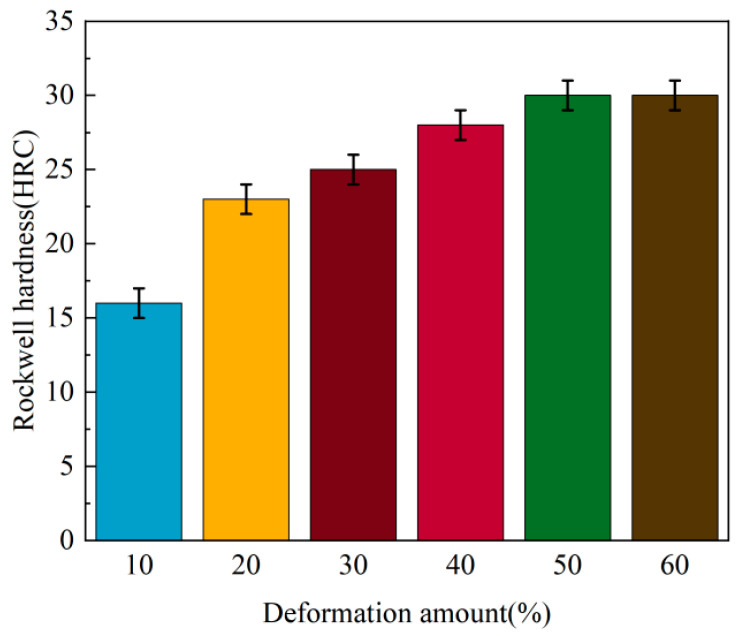
The influence of cumulative deformation on hardness.

Figure 3 shows the influence of cold-drawing deformation on Rockwell hardness. When the cumulative deformation amount is 10%, the hardness is 16 HRC. As the cumulative deformation amount increases, the hardness increases rapidly; when the cumulative deformation amount reaches 30%, the hardness reaches 25 HRC. Notably, when the cumulative deformation amount exceeds 30%, the effect of hardness improving with the increase in deformation amount is significantly weakened, as indicated by a marked decrease in the upward trend of hardness. Eventually, when the cumulative deformation reaches 50%, the increase in hardness is very small, while when it reaches 60%, the hardness remains unchanged at 30 HRC.

**Figure 4:**
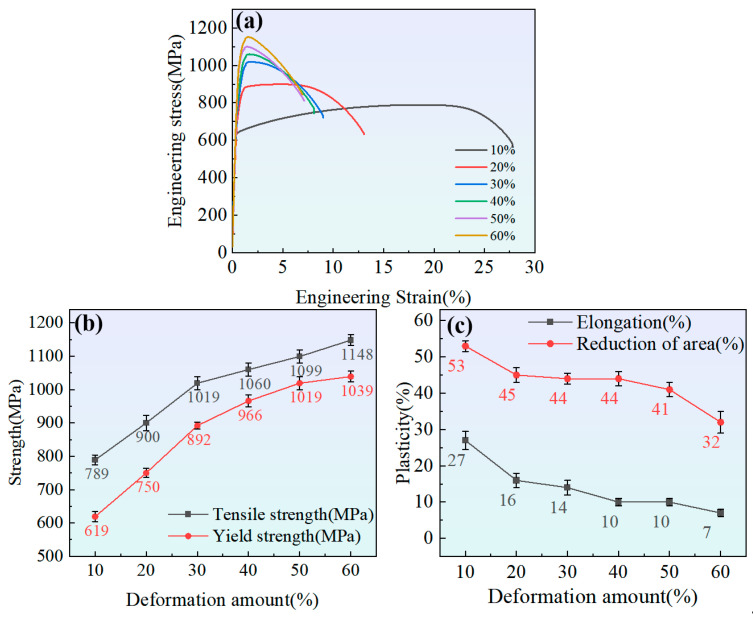
Effects of cumulative deformation on room-temperature tensile properties. (**a**) Room-temperature tensile engineering stress–strain curves; (**b**) tensile and yield strength at different cumulative deformation amounts; (**c**) elongation and reduction in area at different cumulative deformation amounts.

Figure 4a shows the engineering stress–strain curves of samples with different cumulative deformation amounts at room temperature. As the strain increases, the ultimate tensile strength corresponding to the peak of the curve gradually rises while the elongation gradually decreases. The curve has no obvious yield point. Figure 4b shows the values of tensile and yield strength versus deformation amount, demonstrating that as the cold-drawing deformation amount increases, the tensile and yield strength increase rapidly; however, after reaching 30%, the strength increase rate slows down, which is consistent with the change in hardness. When the deformation amount reaches 60%, the tensile strength and yield strength are 1148 MPa and 1039 MPa, respectively. Figure 4c shows the influence of the cold-drawing deformation amount on elongation and reduction in area; as the deformation amount increases, the elongation and reduction in area gradually decrease. When the deformation amount reaches 60%, the elongation and reduction in area are 7% and 32%, respectively.

### 3.3. Microstructural Evolution

**Figure 5:**
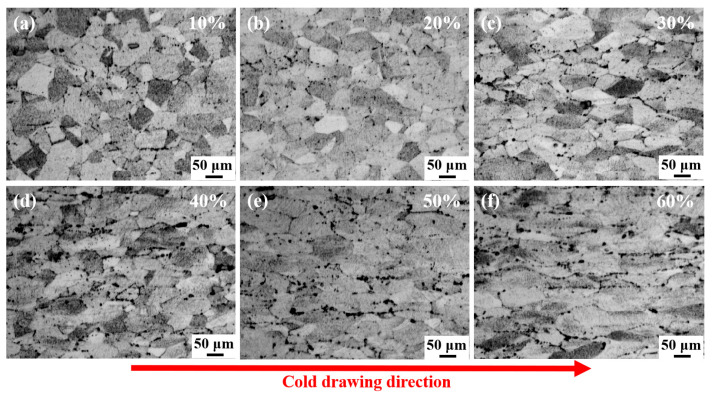
The influence of cumulative deformation on microstructure. (**a**) 10%; (**b**) 20%; (**c**) 30%; (**d**) 40%; (**e**) 50%; (**f**) 60%.

Figure 5 shows the law of influence of cumulative deformation on the microstructure. It should be noted that the distribution of grain size presents non-uniformity, and there is a significant difference between that at the surface and in the interior, which has a certain impact on the overall performance of the alloy.

The red arrow in Figure 5 indicates the cold-drawing direction. As the deformation amount increases, the microstructure undergoes significant changes, and the grain shape gradually changes. Under the tensile stress along the drawing direction, the grains gradually transform from equiaxed to flattened. When the deformation amount reaches 50%, the grains are severely elongated and even split. Moreover, the grain boundaries gradually become unclear, presenting a fibrous structure.

## 4. Discussion

### 4.1. The Influence of Cold-Drawing Deformation on Microstructure

**Figure 6:**
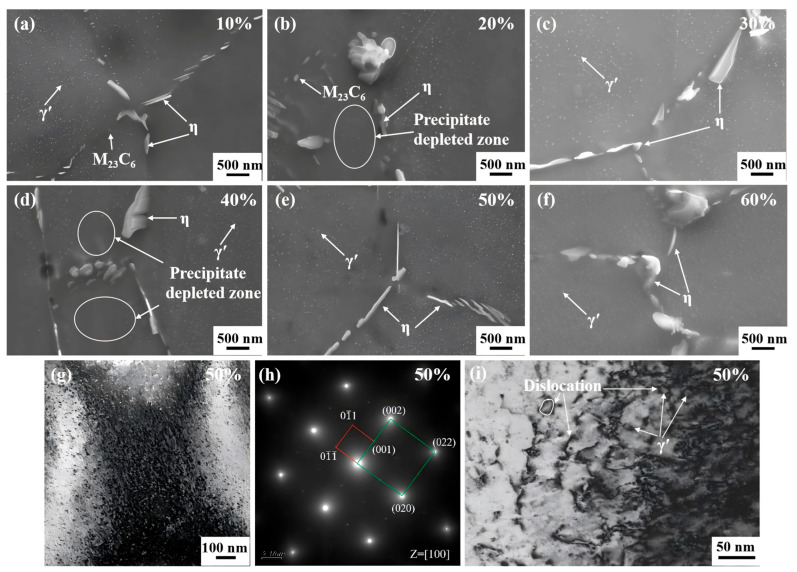
The influence of cumulative deformation on precipitates: (**a**) 10%; (**b**) 20%; (**c**) 30%; (**d**) 40%; (**e**) 50%; (**f**) 60%; (**g**) dark-field transmission electron microscopy (TEM) image with 50% deformation; (**h**) transmission electron microscopy–selected area electron diffraction (TEM-SAED) diffraction pattern with 50% deformation; (**i**) mechanism diagram of 50% deformation of γ’ phase hindering dislocation motion.

**Figure 7:**
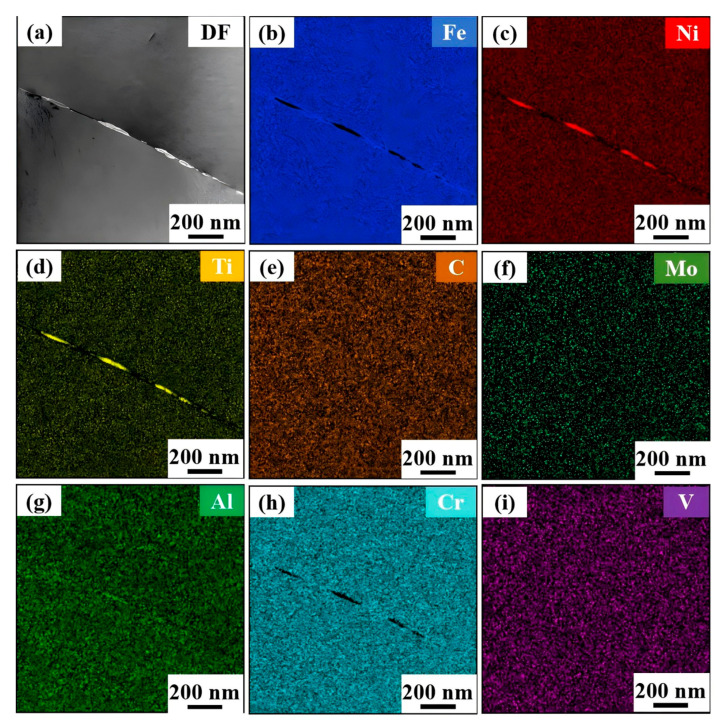
Transmission electron microscopy–energy-dispersive X-ray spectroscopy (TEM-EDS) image of 50% cumulative deformation grain boundary precipitates. (**a**) DF diagram of precipitates at grain boundaries; (**b**) Fe; (**c**) Ni; (**d**) Ti; (**e**) C; (**f**) Mo; (**g**) Al; (**h**) Cr; (**i**) V.

To further investigate the mechanism by which cold-drawing deformation influences microstructure, SEM, EBSD, and TEM were conducted. Figure 6a–h show the microstructures of specimens with cumulative deformation amounts ranging from 10% to 60%, demonstrating that there are numerous spherical precipitates dispersed within the grains of all six specimens with different cumulative deformation amounts. The rod- and block-shaped precipitates are distributed at the grain boundaries. The compositions of precipitates at the grain boundaries of the specimens with 10% and 20% deformation amounts were measured via scanning electron microscopy–energy-dispersive X-ray spectroscopy (SEM-EDS), with the results shown in Table 2, confirming that the small-sized precipitates at the grain boundary are M_23_C_6_ phase. Figure 6g,h show the dark-field morphology and transmission electron microscopy–selected area electron diffraction (TEM-SAED) image of the sample with 50% cumulative deformation. Electron diffraction imaging was conducted along the [100] crystal zone axis. From the diffraction spots, both the matrix and nano-precipitate possess a face-centered cubic structure, while the nano-precipitates possess an L1_2_ ordered structure. By comparison with the standard γ’ phase card, it is determined that these nano-precipitates are of the γ’ phase [35]. As a purely mechanical deformation method, cold drawing mainly stores energy through dislocation proliferation and lattice distortion, and cannot directly provide the driving force to form precipitates. A possible reason for this is that these precipitates are inherent characteristics of hot-rolled materials. After annealing at 850 °C for 0.5 h, the γ’ phase is not completely dissolved and is inherited into the cold-drawn material. The sizes of the γ’ phases with different cumulative deformation amounts are basically the same, with an average size of 36 nm, as measured by ImageJ 1.47 software. However, the precise composition of the long strip-shaped precipitates at the grain boundaries cannot be determined through SEM; therefore, the transmission electron microscopy–energy-dispersive X-ray spectroscopy (TEM-EDS) detection method was employed for examination, and the results are presented in Figure 7. The long strip-shaped precipitates are primarily composed of three elements, Ni, Ti, and Al, confirming that they are of the η phase. With the increase in accumulated deformation amount, the size and morphology of the γ’ phase, M_23_C_6_ phase, and η phase do not change significantly. In addition, in Figure 6b,d, the existence of precipitate-depleted zones is observed, indicating that the formation of the η phase consumes a large amount of nearby Ni, Ti, and Al elements, resulting in a significant reduction in the γ’ phase and the formation of precipitate-depleted zones. Figure 6i shows the hindering mechanism of relative dislocation motion of γ’ in a 50% deformed alloy rod, demonstrating that the γ’ phase hinders the extension and expansion of dislocations. The larger and nondeformable γ’ phase causes dislocations to bend under external force, bypassing the γ’ phase and forming Orowan dislocation loops. The local stress field generated by the accumulation of dislocations around the γ’ phase further hinders the subsequent movement of dislocations, forming multiple strengthening effects.

**Figure 8:**
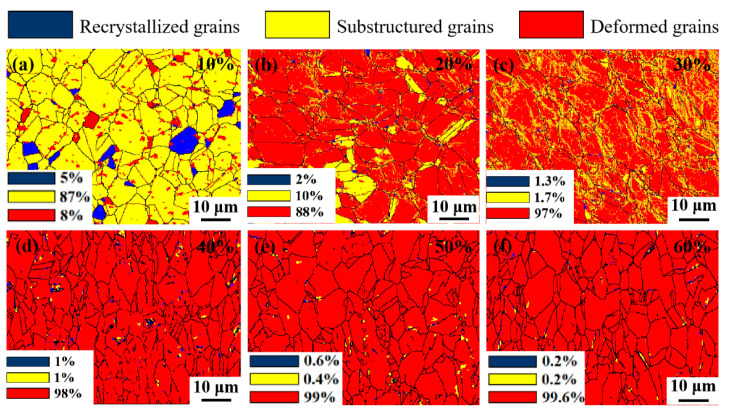
Evolution diagram of deformation of Fe-25Ni-15Cr alloy grains under different cumulative deformation amounts (recrystallized grains (GOS ≤ 1.5°, blue), substructured grains (1.5° < GOS ≤ 5°, yellow), and deformed grains (GOS > 5°, red). (**a**) 10%; (**b**) 20%; (**c**) 30%; (**d**) 40%; (**e**) 50%; (**f**) 60%.

Figure 8 shows the deformation grain evolution of Fe-25Ni-15Cr alloy under different cumulative deformation amounts. The grains were classified based on grain orientation spread (GOS) into three types: recrystallized grains (GOS ≤ 1.5°, blue), substructured grains (1.5° < GOS ≤ 5°, yellow), and deformed grains (GOS > 5°, red). Figure 8a shows the 10% cumulative deformation cold-drawing recrystallization diagram, wherein only 8% of the grains are involved in the deformation, with a large number of substructures. Figure 8b shows the 20% cumulative deformation cold-drawing recrystallization diagram, demonstrating that the volume fraction of deformed grains increases sharply, with approximately 88% of the grains deformed and 10% of the grains not participating in the deformation. When the cumulative deformation amount increases to 30%, as shown in Figure 8c, almost all grains participate in the deformation. With further increases in the cumulative deformation amount, the grains deform more completely; after the deformation amount reaches 60%, the proportion of deformed grains reaches 99.6%, as shown in Figure 8f.

**Figure 9:**
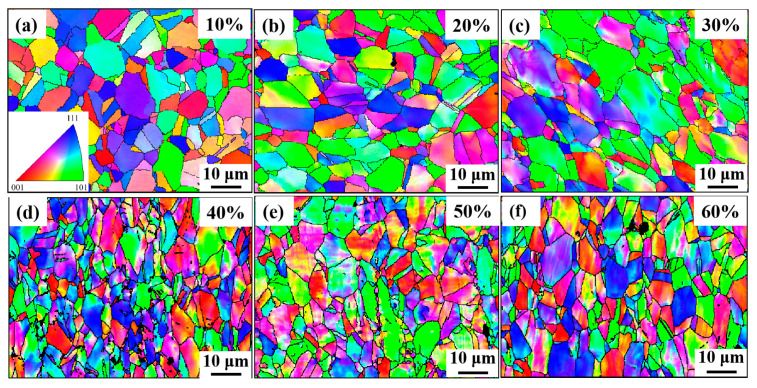
Inverse pole figure (IPF) map of different cumulative deformation amounts. (**a**) 10%; (**b**) 20%; (**c**) 30%; (**d**) 40%; (**e**) 50%; (**f**) 60%.

Figure 9 shows the inverse pole figure (IPF) of different cumulative deformation amounts, demonstrating that the 10% cold-drawing deformation grains are basically equiaxed, and the grain orientations are approximately random, with no obvious preferred distribution. For the Fe-25Ni-15Cr alloy with a face-centered cubic structure, the [101] orientation only has four equivalent {111} <110> slip systems, which are few in number and have relatively high resolved shear stress, resulting in poor deformation coordination ability. However, although the [001] orientation has a relatively high resolved shear stress, there are eight equivalent slip systems, and the probability of dislocation cross-slip is high; thus, it possesses good deformation coordination ability [36]. In this experiment, the volume fraction of grains with different orientations was calculated using a tolerance of 15°. Due to the difficulty of deforming [101]-oriented grains, their volume fraction did not show significant regular changes during the 10% to 50% deformation process, and remained stable at around 16.5%. After the deformation reached 60%, the volume fraction of oriented grains decreased sharply to 9.6%, though it showed an overall upward trend, and during the process of increasing deformation from 10% to 60%, increased from 5.43% to 13.1%. The volume fraction of oriented grains also showed an increasing trend: the mechanism volume fraction of this orientation increased from 12.8% to 18.2%, and the [111] orientation is usually the final stable component of the texture in stretched FCC metals. This is because the sliding direction of the grains along the [111] orientation is symmetrically distributed relative to the axial direction, which helps to maintain the circular cross-section of the metal wire during the cold-drawing process. In addition, the [111] texture can promote the formation of twins and enhance the strength of the hard orientation [30,37,38,39].

### 4.2. The Influence of Cold-Drawing Deformation on Dislocations

**Figure 10:**
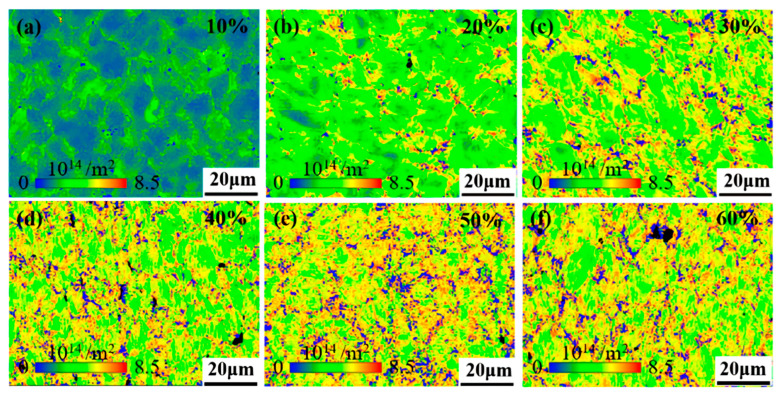
Local dislocation analysis of different cumulative deformation amounts. (**a**) 10%; (**b**) 20%; (**c**) 30%; (**d**) 40%; (**e**) 50%; (**f**) 60%.

**Figure 11:**
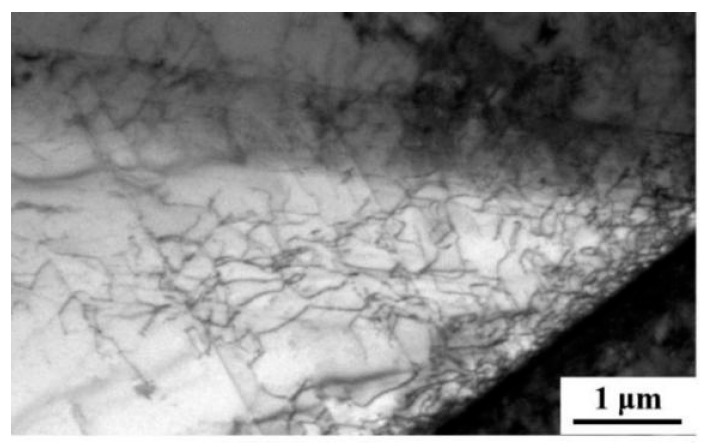
Local dislocation analysis at 50% cumulative deformation.

A GND density map is obtained by quantitatively calculating the nuclear mean orientation error using a square kernel with a size of 5 × 5 pixels^2^, and excluding data points with orientation error values greater than 5° from the analysis. Figure 10 shows the GND maps of Fe-25Ni-15Cr alloy after cold drawing. The scale shows that the dislocation density gradually increases with the gradual increase in the cold-drawing deformation amount. Through calculation, the dislocation densities for six cumulative deformation amounts ranging from 10% to 60% are 0.19, 0.30, 0.43, 0.49, 0.52, and 0.52 × 10^14^ m^−2^, respectively, demonstrating that as the deformation amount increases, the growth rate of dislocation density gradually slows down. After the cumulative deformation amount reaches 50%, the dislocation density basically tends to saturate. That is to say, when the deformation amount reaches 50%, further cold drawing will not increase the work hardening effect. In combination with Figure 11, it can be seen that the dislocation density is preferentially accumulated at the grain boundaries and gradually extends into the grains with the increase in deformation amount, forming a cellular substructure.

### 4.3. The Influence of Microstructure Evolution on Mechanical Properties

During the cold-drawing deformation process, the grains will be rearranged and oriented to adapt to the new shape and size. This process of grain refinement and microstructure optimization enhances the hardness of the alloy. Additionally, dislocations first occur at the grain boundaries in austenite, and at this stage, the distribution of dislocations is relatively uniform in a random state. As the deformation amount gradually increases, the dislocation density increases, entangles at the grain boundaries, and gradually extends into the grains. The dislocation density at the grain boundaries is higher than that within the grains [40,41].

Further increasing the deformation amount leads to a faster proliferation of dislocations and a more uneven distribution. Disordered dislocations gather together to form high-dislocation-density regions with dislocation entanglements, which are separated from the areas with low dislocation density, thus creating a cellular substructure. Dislocation density is very high at the cell walls, while it is very low inside the cells. The initially formed cellular structures are basically equiaxed; as the deformation amount increases, some local areas experience uneven deformation and form non-equiaxed cellular structures. These structural dislocation pinning effects can significantly enhance strength and hardness while reducing plasticity. When the deformation reaches 30%, the increase in strength and hardness slows down significantly, which corresponds to the changes in grain size and dislocation density mentioned earlier. The grains are stretched to their limits, causing the grain boundaries to break and forming fibrous stripes distributed along the stretching direction [42,43]. The grain boundaries become blurred or even disappear, and the dislocation density also tends to reach a saturated state. The Hall–Petch strengthening effect and work hardening effect are no longer enhanced, causing the increase in hardness to slow down significantly, and when the deformation exceeds 50%, the increase in hardness nearly ceases [44,45].

In addition, the cold-drawn billets used in this experiment were subjected to annealing treatment before cold drawing, resulting in the precipitation of some γ′ phases. The γ′ phase is the main strengthening phase of Fe-25Ni-15Cr alloy, exerting a significant impact on its strength; therefore, the strength contribution is the combined effect of fine grain strengthening, work hardening, and second-phase strengthening. Based on the work hardening law, Hall–Petch relationship, and Orowan strengthening model and through substituting the initial tensile strength of 673 MPa and grain size of 12.98 μm, the final model of tensile strength is obtained as follows:

(1) $$\sigma_{y}=712+320\varepsilon^{0.6}+173\sqrt{1+0.314\varepsilon }$$

The prediction results of the model are shown in Table 3. The average error is 6.9%, and when the cumulative deformation is 10% and 20%, the errors are 22.6% and 12.4%, respectively. When the cumulative deformation amount reaches 30%, the error is 2.8%, and the accuracy is significantly improved. As the cumulative deformation amount increases, the error continues to decrease and eventually stabilizes at around 1.0%. The errors for the cumulative deformation amounts of 10% and 20% are relatively large, because a large number of grains do not participate in the cold-drawing deformation under small deformation amounts, resulting in relatively large errors in the calculation results regarding fine grain strengthening and work hardening.

## 5. Conclusions

In this study, we conducted experiments on Fe-25Ni-15Cr alloy bars prepared by vacuum induction melting, electroslag remelting, forging, hot rolling, and annealing processes with different cold tensile deformation amounts (10% to 60%) and systematically investigated the effects of these different deformation amounts on their microstructure evolution and mechanical properties. We draw the following conclusions:

(1) When the cumulative deformation amount reaches 50%, the grains transform from equiaxed to fibrous structures; dislocation density accumulates preferentially at the grain boundaries and forms cellular substructures, while the texture orientation gradually stabilizes from random distribution to the <111> direction.
(2) Fe-25Ni-15Cr alloy bars fabricated via vacuum induction melting, electroslag remelting, forging, hot rolling, and annealing processes exhibit three strengthening mechanisms during cold drawing: grain refinement strengthening, second-phase precipitation strengthening, and work hardening.
(3) When the deformation amount is less than 30%, the hardness, tensile strength, and yield strength increase significantly with the increase in deformation amount, while the plasticity and toughness continue to decline. This is mainly attributed to the increase in dislocation density, grain refinement, and the formation of cellular substructures. However, when the cumulative deformation amount exceeds 30%, the grain vibration and dislocation density tend to saturate, resulting in a weakened strengthening effect and a significant decrease in the rate of increase in hardness and strength.
(4) The prediction model of the cold drawing and tensile strength cumulative deformation amount is obtained through experiments and theoretical calculations: $\sigma_{y}=712+320\varepsilon^{0.6}+173\sqrt{1+0.314\varepsilon }$.

## Figures and Tables

**Table 1:** Chemical composition of Fe-25Ni-15Cr alloy (wt.%).

Elements	C	Fe	Ni	Cr	Mo	Co	Ti	Al	V
Content	0.05	53.90	24.56	15.12	1.26	0.20	1.79	0.19	0.32

**Table 2:** Results of scanning electron microscopy–energy-dispersive X-ray spectroscopy (SEM-EDS) analysis.

Elements (at%)	C	Fe	Ni	Cr	Mo	Ti	Al
10% deformation amount	20.66 ± 3	41.20 ± 2	17.50 ± 1	11.12 ± 0.5	0.62 ± 0.1	1.91 ± 0.1	0.64 ± 0.1
20% deformation amount	20.80 ± 3	40.50 ± 2	18.90 ± 1	12.30 ± 0.5	0.60 ± 0.1	2.04 ± 0.1	0.60 ± 0.1

**Table 3:** Experimental and model prediction results for different cumulative deformation amounts.

*ε*	Actual Value (MPa)	Model Predicted Value (MPa)	Error (%)
0.1	789	968	22.6
0.2	900	1012	12.4
0.3	1019	1048	2.8
0.4	1060	1080	1.8
0.5	1099	1109	0.9
0.6	1148	1136	1.0
Average error			6.9

## Data Availability

Data are contained within the article.
